# Super-Oxygenation as a Therapeutic Measure, with Notes of Cases Treated

**Published:** 1869-03-01

**Authors:** S. S. Wallihan

**Affiliations:** Chicago


### *Super-Oxygenation as a Therapeutic Measure, with Notes of Cases Treated.*

BY S. S. WALLIHAN, M. D., CHICAGO.

[Concluded.]

All the metallic per-oxides give off oxygen, when heated to redness; and no doubt, eventually, the disengagement of the gas will involve only the expenditure of a certain amount of heat.

In emergencies, and when a prolonged use of the remedy is not required, any of the foregoing, or equally unobjectionable methods may be used. When exhibited in chronic cases, involving protracted use of the remedy, and in some cases of unusually sensitive lungs, or of repugnance to the slightest disagreeable taste or smell, only those processes which afford the gas in a state of the utmost purity are admissible.

The apparatus for the use of condensed gas in iron bottles, invented by Barth, of London, although very convenient for such practitioners as find it out of the question to manufacture their own gas, and possessing some decided advantages for all, has never come in much request.

Galante, of Paris, has perfected a very convenient apparatus for the manufacture of pure oxygen, but its expensiveness, including delay and cost of importation will preclude its use in this country. Dr. Beigel has patented a form of apparatus and inhaler, which is both competent and convenient. Sprague, of Boston, has brought an apparatus which possesses some merit, in having attached a polished, patented, automatic heat-regulator; but I do not consider its advantages sufficient to warant the somewhat extravagant price asked for it.

I am satisfied, however, that *the short-comings of impure and carelessly manufactured gas have done more than all else to bring the treatment into discredit, and prevent its use by the profession at large.*

1. *Protoxide of nitrogen*, the real value of which, as a therapeutic, has been overshadowed by its brilliant properties as an anæsthetic. More than twenty years ago, Dr. Zeigler, of Philadelphia, experimenting with it, for dental purposes, also administered it, tentatively, in moderate doses, for chronic general torpor of the system, nervous and other functional disorders, with very encouraging results. In his work on this gas, now some years out of print, he dwells on its physiological action, when exhibited to proper subjects in suitably regulated doses, is very positive in his conclusions as to its therapeutic value, and urges the medical profession to recognize and further develop its claims as a remedial agent. His deductions were, of course, entirely empirical, at least he made no attempt to elucidate the *rationale* of its action.

Dr. Barker, a more recent authority on the same subject, quotes Dr. Zeigler, as above, and coincides with him.

Dr. MacCormack thought that, properly diluted, it would be found valuable in the treatment of some forms of disease; but he seems not to have used it to any extent, and predicated his opinion wholly on the proportion of oxygen contained in the compound.

Garrod, and other recent authors on Materia Medica, do not mention it.

Dr. Hartshore, of Philadelphia, in his *Essentials of Praccal Medicine*, (Phila., 1868,) advises a trial of it in bad cases of asthma, evidently with a view solely to its anæsthetic effects. Dr. Birch doubts if it be in any sense a super-oxygenating agent. Other authorities on the medical uses of oxygen, make no mention of this compound.

"Oxygenized Air," of late so over-lauded in advertisements, is nothing more or less than a certain admixture of protoxide of nitrogen with free oxygen and common air. In fact, in most instances, as administered by several pseudo-prophets each of whom claims to be a "discoverer," and has been selling "rights" to use the treatment, it is composed of protoxide only, given in moderate doses.

Used alone, aside from its anæsthetic properties when exhibited in larger doses, I do not attach so much importance to this agent as some others have done; but as a modifier of pure oxygen I consider it invaluable. In some spasmodic affections I have found its use a valuable substitute for chloroform and the ethers. It often succeeds when these utterly fail, and it is never followed by the disagreeable and some times serious symptoms attending their exhibition. It is excellent in neutralizing affections, and in certain forms of "nervous headaches" it has proved almost a specific. I have invariably succeeded in overcoming the wakefulness of nervously debilitated women by the administration of moderate doses at bedtime.

Its greatest value, however, as it appears to me, lies in in its use as a diluent and vehicle for pure oxygen. Without venturing upon any discussion of its chemical or physiological action on the human organism, reference to a few known facts concerning it will be in place:

First, it is the most soluble of all gases, water at ordinary temperatures, absorbing its own volume of the gas.

Second, it supports combustion almost as perfectly as oxygen itself.

Third, the exhilarating properties popularly ascribed to it have been very much over-stated, a majority of those inhaling the pure gas experiencing nothing of the kind.

Fourth, its effects on the nervous system are peculiar, and may be made so slight as to be inappreciable, or so powerful as to produce momentary insensibility, with any desired degree of approximation to either extreme.

Fifth, inasmuch as it is largely absorbed by the system, when taken into the lungs, and produces many of the same effects as follow the use of oxygen itself, which it also very much resembles in its action on metalic and other oxidizable substances, it may be safely assumed that it is, of itself, a powerful super-oxygenating agent.

But, setting aside its claims as a therapeutic *per se*, I have become fully satisfied that, used as a modifier of oxygen gas, it plays no unimportant part *as an adjuvant*, while at the same time *increasing the solubility of the basic element.*

In this connection I desire to call attention to a point which seems to have escaped the attention of every author who has written on the use of oxygen.

It is well known that alcohol is the basis of all, so called, "spirituous" beverages; but no physician, desiring to exhibit a stimulant, prescribes raw alcohol, or alcohol and water. Choice brands of old wines, brandies, whiskys, etc., although utterly worthless without it, are not valued according to the percentage of spirits they contain; and *the action of oxygen, properly "toned down" and modified is as different from its action in an unmixed and undiluted state as is the action of absolute alcohol from that of the best French brandy, or good grape wine.* From my repeated tests of the gas, in every form of administration, and in a great variety of cases, I am forced to to the conclusion that, except in certain comparatively rare contingencies, and then, as necessarily, only for temporary purposes, *unmodified oxygen gas is unfit for general therapeutic use as is unmodified and undiluted alcohol for use as a beverage.* Nor do I consider its dilution with common air, as practiced by Goolden, Birch and others, better, in comparison, than alcohol for medicinal use, diluted with simple water.

Not to discuss this point further, I am persuaded that another source of some reputed "failures" of the oxygen treatment, has been from the neglect of experimenter to properly modify the powerful agent (for good or evil) they have tampered with.

3. *Oxgenated water*, probably the best vehicle for the introduction of oxygen into the system by means of the stomach, is prepared by converting water into steam, thus expelling all its atmospheric air, after which pure oxygen gas is brought in contact with the steam, under high pressure, and takes the place of the expelled air. The water thus becomes a saturated solution of the gas, and is the only oxygenated water worthy of name. At present it can be procured only in England, where it is prepared with considerable care by the Messrs. Robbins & Co., and by Barth, of London. Dr. Barth was first to call attention to it, and has patented an apparatus for its manufacture. It is to be hoped some enterprising manufacturing chemist, in this country, will undertake its careful and thorough preparation. Whenever direct absorption into the gastric and portal circulation is the object in view, this is a most eligible form for the exhibition of the remedy. To what extent it may possess antidotal and antiseptic properties, in some forms of zymotic diseases, *e. g.*, diphtheritis, cholera, scarlatina, etc., remains to be demonstrated. I anticipate important results in this direction. The advantages of ready preparation and portability render it especially valuable in the absence of, or in conjunction with other methods of administration.

4. *Nitrous-oxide water*, properly prepared, will, in time, become more popular than now.

While considering it far inferior to *oxygenated water*, Dr. Birch admits that "in some nervously depressed and hypochondrical patients" he would give it the preference. It was originally put forth as a patented nostrum, and had quite a sale; but it may be questioned whether some of its properties are not measurably due to the trace of nitrous acid generally present.

5. *Oxygenated bread* is being manufactured and used, to some extent, in England, and is highly extolled by several practitioners of some repute. Dr. Birch, in particular, is quite sanguine respecting its claims, predicting that it will prove a boon in medical practice. It is prepared on the same principle as oxygenated water, *i. e.*, by substituting pure oxygen gas for common air and carbonic acid, in aèrating the dough. Processes have been perfected in its manufacture by which it is made to retain its own sweetness and preserve the perfect occlusion of the oxygen for several weeks.

"A surprisingly small bit of this bread proves its special value *in suitable cases.* * * * Its relative effect on the appetite is singular. Thus, on the one hand (as might be expected) it stimulates the appetite when absent or capricious, while, on the other hand, it tends to produce such a feeling of epigastric fullness, when sufficient food has been taken, as to effectually suspend (if not satisfy) some morbidly craving appetites. * * * *In some cases* it will be found advantageously to supersede artificial, pepsine, pancreatine, and even quinine and the mineral acids. * * * It is particularly indicated in cases of delicate children with continually recurring ascarides, functional derangements of stomach and bowels, and mesenteric weakness." (Birch op. cit., pp. 18, *et seq.*)

If the value of oxygen, when exhibited by the stomach, be fully established, then I have no doubt oxygenated bread, made with sufficient care and accuracy, is a desirable medium for its administration, and may be found a desideratum in such cases as referred to by Dr. Birch, or where, for any reason, inhalation is objectionable or impossible.

6. *Per-oxide of hydrogen*, in solution, has been recommended for internal use, by Dr. Richardson and others. Dr. Goolden, of St. Thomas' Hospital, has used it, but finds it much less reliable and efficient than other preparations, as well as somewhat objectionable on account of it disagreeable flavor. It must be borne in mind that this compound is a very unstable one and liable to explosive decomposition.

Of other preparations, whose claims as oxygenating agents, have been urged, may be mentioned—*ozone water*, which is evidently but another name for an inferior article of oxygenated water, but for which special virtues have been claimed by a few practitioners. As brought to my knowledge, it is simply water through which oxygen gas, made by the ordinary chlorate of potash and manganese method, has been passed for purification. The water absorbs but about four or five per cent. by volume, of the gas, but contains also traces of chlorine, to which element the attractive, antiseptic and *anti-syphilitic* properties ascribed to it, may be largely due.

*Ozonized oil* has nothing but its disagreeableness to recommend it; hence it has acquired the name of " rancid oil."

The *permangauates*, *perchloric*, *nitric* and *hydrochloric acids* and the *chlorate of potash*, are all, undoubtedly, sources of free oxygen, when internally administered. Each of these is rich in oxygen and readily yields up one or more equivalents, under slight modification of conditions; hence the inquiry is entirely pertinent, as to how far *the tonic*, *antiseptic and other effects ascribed to these remedies, is due to the free oxygen evolved during their decomposition and solution in the system.*

In a majority of cases, I have used the remedy chiefly in gaseous form, administered almost exclusively by inhalation. In several, I have tentatively used oxygen gas; first, pure and of full strength; second, diluted with common air; and third, admixed in various proportions with protoxide of nitrogen and common air.

In severe paroxysms of spasmodic asthma, which have continued until the system seriously suffers from non-aëration of the blood and general veinous congestion, in cases of asphyxia, fatal syncope, and other cases of suspended animation, and in some other emergencies—*e. g.*, threatened uræmic poisoning—the first form of administration may be selected as the best. For general use, and in ordinary cases, the last form mentioned is decidedly preferable. The second form may be substituted when the gas, in its full strength, is not required or admissible, and the protoxide is not available.

In the matter of proportions, and as respects the quantity to be used in various cases, there is as great range for selection and the exercise of professional judgment and discretion as in prescribing and compounding complex pharmaceutical preparations. Those physicians who think they "know all about the oxygen treatment,"—and they are not a few—having at some time administered, or, as is too often the case, having only *read* of the administration by inexperienced parties, of a few gallons, more or less, of what might be termed "varo gas," have blundered as stupidly and failed as utterly, as he would who should prescribe a few desultory doses of crude drugs, and then *pro*scribe the remedies if they failed to produce satisfactory constitutional effects. I may add, that I have often heard the use of oxygen abruptly condemned by physicians, but in no instance by one who has had the slightest practical knowledge of its effects.

The reply of an eminent English surgeon is suggestive. When asked his opinion of the treatment, he candidly admitted that, "having never administered it himself nor witnessed its administration in the hands of others, he could express no intelligent opinion concerning it."

That it requires some expenditure of care and time in its manipulation, and the exercise of much discretion in its exhibition, and that some practitioners will fail in its use, through lack of these essentials, is very true; but that it adds so largely to our therapeutic resources—is, in fact, a desideratum in so many cases and contingencies, that no conscientious physician, who has once realized its thoroughly scientific claims, and witnessed its intelligent administration, will neglect to avail himself of its aid in all suitable cases.

I append brief clinical notes of a few selected cases in which I have met with marked success through the use of some form of super-oxygenation:

Case 1.—Mrs. J. S———, aged thirty-nine, by profession a singer and teacher of vocal music, gives the following history:

Several of her family have died of phthisis, and for several years past she has shown symptoms of its gradual invasion. Each winter she is troubled with severe bronchitis. It is now worse than ever before, and she has been obliged to forego the use of her voice. She is alarmed lest her present condition is premonitory of rapid and fatal decline. "It is just as the others started." I find her pale, anæmic, with rapid, weak pulse, cold extremities, severe cough and sub-clavicular pain, accompanied by a profuse muco-purulent expectoration. She is a confirmed dyspeptic, and as is usual in such cases, suffers greatly from functional derangement of the heart. Her appetite is poor and capricious, bowels constipated, and she is excessively fatigued on slight exertion. She is despondent, hysterical, and only consents to a trial of oxygen treatment at the earnest solicitation of her friends.

March 25, 1868, she presents herself for treatment. At a forced inspiration she can inhale 150 cubic inches of the gas, which I adapt to her peculiarly prostrate nervous condition by larger additions of protoxide of nitrogen.

April 1st.—The nervous depression of this patient is almost entirely relieved. She coughs no less—rather more, and expectorates more freely. She continues to take treatment once daily, and can now inhale 180 cubic inches at an inspiration.

April 10th.—Improvement in every respect is very marked. Her appetite and spirits have returned—she coughs less, and her expectoration has diminished, and lost its purulent character; extremities comfortable, *constipation relieved*, and she is enabled to resume her place in the choir, having no trouble to do even double duty with her voice.

April 20th.—The improvement continues. Her general health is better than for a long time. The bronchial trouble has disappeared, she has no cough, spirits never were better—has no thoughts of dying of "consumption;" sings, works and *eats* without fatigue or distress. Has gained in flesh, her cheeks bloom with healthy color, and she has increased her lung capacity to 220 cubic inches.

April 25th.—Patient considers herself *well*; appetite and digestion excellent, pulse full and strong, no more palpitation—she is as happy and hopefu as she previously was wretched and despondent.

Treatment suspended. (I have met this patient frequently since, and there has been no return of the untoward symptoms. Jan. 1869.)

Case 2.—May 23d. Mrs. C. L. W———, aged twenty-nine, married, applied for treatment. Has always had "weak lungs," and her friends fear the development of pulmonary disease. Her father died of cancer of the stomach, mother of cardiac disease (?) Has had a severe cough and circumscribed "sore spot," with a sense of constriction in her left lung, for a long time. Has been under the care of several eminent physicians, without benefit.

She is "nervous" and anæmic. The menstrual function has not been properly performed in three years; is often entirely wanting—at times the discharge is profuse, acrid and colorless; at others it is scanty, very offensive, and of a greenish or yellow color. She also suffers from frequent attacks of severe congestion of the kidneys, and has, almost constantly, a sense of weight and soreness in the region of these organs.

Placed her upon moderate doses of oxygen, well diluted with protoxide and common air.

The first dose affords a degree of immediate relief. She feels "rested," and the sense of "tightness" in her chest is lessened. Lung capacity 140 cubic inches.

June 1st.—Improvement has been constant and gratifying. Appetite and digestion have become perceptibly better. She coughs less, and the sense of soreness is scarcely perceptible; is buoyant and hopeful, and has a tinge of color in her cheeks. Inhales 165 cubic inches, and I order her to inhale moderate doses, twice daily, for a time.

June 15th.—The usual complication has apparently assumed a critical form—what were chronic becoming sub-acute conditions. She suffers considerably and is greatly alarmed. Active oxygen treatment suspended for the present, and measures of a soothing nature are addressed to the local malady—hot fomentations over the regions of the kidneys, and a hot hip-bath twice daily, with some other derivative measures, are prescribed, and moderate inhalations of dilute protoxide alone are administered.

June 20th.—The acute manifestations noted on the 15th soon subsided, and now the patient feels better than for years. Treatment resumed, with an increase in the proportion of free oxygen, and the quantity taken twice daily, somewhat diminished. She can now inhale 200 cubic inches at an inspiration.

June 25th.—Improvement since last record quite marked, and patient very sanguine of ultimate restoration to a fair degree of health.

July 1st.—Menstruation natural for the first time—painless, the discharge normal in quantity and of healthy color. Patient no longer "nervous"—has considerable color in her face, and walks from two to three miles daily without fatigue. Her voice, which had failed her in singing, is again strong, the soreness and constriction entirely gone—coughs but little, eats heartily, digests well, sleeps better than she can remember to have done since childhood, and has gained considerably in flesh. She has developed her lung-capacity to 225 cubic inches, and her chest measurement accordingly. Her friends are greatly surprised at her improved appearance.

July 10th.—Patient continues to improve. Treatment moderated, and conjoined with a mild chalybeate course.

July 25th.—Treatment suspended, and patient returns to her friends, feeling that she has a stronger hold on life than she had dared to hope was attainable. With proper care and under favorable conditions, she is in a fair way to live many years, and enjoy a fair degree of health.

Case 3.—Miss A. B.———, aged nineteen, has been subject to severe paroxysms of asthma, quite frequent, for some years. A taint of the disease seems to be hereditary. The slightest changes of temperature and exposure bring on a paroxysm, which lasts from one to six days, and is often so violent as to alarm her friends. Even the inhalation of chloroform fails to control the spasm, when it is severe.

She has changed climate several times, by the advice of physicians, and has recently consulted and received a course of treatment at the hands of a noted throat-and-lung "specialist."

Nothing she has ever done having given her any permanent relief, she was brought by her father, Judge B———, to the office for advice, May 25th.

I found her delicately organized, highly sensitive, quite anæmic, as might be expected, and suffering from evident general venous congestion. There is a dark shade about her eyes, and she is excessively fatigued on slight exertion. I very confidently recommended a trial of the oxygen treatment, and she was placed upon daily inhalations of protoxide, containing but a trace of free oxygen. Lung capacity but 75 cubic inches.

June 4th.—She reports that the gas has "cured" her; she has had no oppression of her breathing since she began it. She has improved considerably in general appearance. Her complexion is as clear as could be wished. She inflates her lungs well and with perfect ease—inspiring 100 cubic inches at an effort.

June 20th.—No return of the paroxysms yet. Her general health has undergone great changes. Formerly she had very little appetite, and that of a morbid and capricious kind. Now she has excellent relish for substantial food, *and wants plenty of it.*

July 1st.—Improvement of the general health continues. Exposure from being caught in a storm, a few days after last record, induced a mild paroxysm of asthma, but it was readily controlled.

July 10th.—Patient feels buoyant and full of courage. Can now endure considerable exercise without excessive fatigue, and her mother reports that "she is really getting to be worth something." Treatment continued every other day. She now takes 140 cubic inches at an inspiration.

July 25th.—Treatment has been somewhat interrupted since last writing; but the general health of the patient continues excellent, and she breathes as freely as as any non-asthmatic. She also exposes herself to changes of temperature with impunity. Does not "take cold" so easily as heretofore,

Aug. 10th.—There has been no return of the paroxysms, although the patient says she has repeatedly exposed herself to the same influences which invariably excited them, previous to treatment.

She is advised to continue the treatment at intervals, but as the patient removed from the city, no further observations are practicable.

(Nov. 30th.—This patient reports that she has continued to enjoy excellent health and has had but one paroxysm since treatment was suspended. She thinks if she could have continued the treatment for a longer period the cure would have have been perfect.)

Case 4.—Aug. 10th.—Miss E. L. C———, aged sixteen, was brought to to me for advice. Has been subject to epileptic convulsions for some years. The family history shows the malady to be hereditary. She is extremely "chlorotic"—tall and slender, with stooping, irregular gait and attitude, feeble pulse, soft, imperfectly developed muscles, sallow complexion, deeply shaded about the eyes, contracted chest, feeble and weak digestion. Has never menstruated. Cephalalgia is a constant symptom; sleep disturbed with horrid dreams, in which she starts, screams, gnashes her teeth, talks incoherently, and is frequently found in the morning hanging across the footboard of the bed, "frothing at the mouth"—evidently having had a paroxysm during the night. I unhesitatingly advised a persevering trial of the oxygen treatment, and

Aug 13th., she was placed upon moderate doses of modified oxygen. Her lungs are extremely small, and imperfectly expanded and she can inhale but 60 cubic inches at one inspiration.

Aug. 20th.—She has perceptibly improved. Her sleep is much more quiet and natural, and there has been no return of the "fits." She can now take 90 cubic inches at an inspiration. Order her to take a liberal and nutritious diet, and place her upon mild chalybeates.

Aug. 30th.—Still no return of the paroxysms, appetite and digestion very much improved, and from being languid and listless, and constantly "tired," she has come to be quite ambitious, and endures considerable muscular exertion without fatigue. Her "headaches" have ceased to annoy her, she sleeps quietly, her complexion has become clear, there is a show of color in her cheeks, and she has gained in flesh. Can inspire 100 cubic inches.

Sept. 14th.—In every respect this patient continues to improve. She attends school again, walking several miles daily, without fatigue.

Treatment continued every other day. Lung capacity 105 cubic inches.

Oct. 1st.—Is becoming quite robust in comparison with her former condition.

Oct. 10th.—From having exposed herself nearly all last night—the evening being quite chilly and damp—in attending a political demonstration, she is, to-day, qnite indisposed, and for an hour or two, manifested symptoms of her former malady. The paroxysm, however, proved slight and readily yielded to ordinary remedies. She is again put upon daily oxygen treatment. Her lung capacity has increased to 115 cubic inches.

Oct. 20th.—No recurrence of untoward symptoms. Seems perfectly well in all respects. Treatment continued every other day.

Nov. 1st.—The patient has continued to gain in flesh and strength. Her appetite is fairly insatiable, and among her mates she is one of the most active. Would be taken at a glance for a more than ordinarily robust person of her age.

Treatment administered but twice a week. She inspires 120 cubic inches.

Nov. 10th.—Her menstrual function has manifested itself naturally, and without unusual disturbance. Her parents are rejoiced, since they have always coupled her convulsions with the absence of the catamenia.

Nov. 25th.—Treatment entirely suspended. Patient in perfect health.

(Jan., 1869, this patient continues in perfect health, enjoying life actively and fully.)

CASE 5.—Sept. 18th.—Mr. A. S———, merchant, aged fifty-one, applied to me. Has been an invalid for twenty years. Has confirmed dyspepsia, and excessive debility aud irritability of the nervous system. Suffers much from constantly recurring paroxysms of a dull, benumbing pain, referred chiefly to the upper portion of the spinal column and base of the brain ; and also has almost constant frontal headache. Constipation is habitual—with occasional attacks of diarrhœa ; appetite poor and irregular, circulation feeble and unbalanced, extremities cold ; almost impossible to keep his feet warm in winter. He is at times extremely irritable, as to mood, and at others is overcome by the most utter depression and despondency. He has suffered considerably from "ague," heretofore, and imagines he feels "dumb ague" now, at intervals. Has long been troubled with a derangement of the kidneys, his urine varying greatly from time to time, but always containing mucous and the phosphates in excess—as he has been informed by physicians who have examined it—his liver and spleen are both enlarged, and his cutaneous exudations have, at times, a very decided ammoniacal odor.

His urine—sample from that first voided in the morning, or urine sanguinis—has a specific gravity of 1030, contains excess of the alkaline phosphates, but there is a marked deficiency of urea and uric acid.

A course of thorough oxygen treatment is advised, with the prediction that several months will be required to effect marked and permanent benefit.

Sept. 20th.—Treatment begun, and the quantity of gas administered increased to double that usually prescribed, since all his vital functions seem oppressed and sluggish, and there is evident retention of urea in the system. Lung capacity 160 cubic inches.

Sept. 29th.—My confidence that oxygen would eliminate any excess of urea from his system, and my suspicions that most of his nervous symptoms arose from such retention, were well grounded. His urine to-day *is loaded with urea.* The nitric-acid test yields a mass of crystals of uric acid *equal to the bulk of both constituent liquids.* Other solids are present in but slight excess. Specific gravity 1035. (This example, like the former one, was from urine voided on first rising in the morning—the patient having taken the unusual quantity of eight gallons of the gas containing three gallons of pure oxygen, at nine o'clock the previous evening.) I could discover no traces of a saccharine or albuminous character.

All his nervous symptoms, which I had attributed to slight uræmic poi. soning, have been materially relieved. He is less irritable, sleeps better and feels encouraged ; but there is considerable disturbance of his general system. He complains of feeling some twinges of an ancient "rheumatism" revived. Treatment moderated by reducing the quantity of gas inhaled to one-half the former dose. He can take 200 cubic inches at an effort.

Oct. 11th.—The uncomfortable sensations and evidences of constitutional disturbance following the rapid treatment of the first week have disappeared. His appetite and digestion are improving. Sp. gr. of urine 1028, with still some excess of urea.

Oct. 18th.—Improvement has been gradual but constant, since last record. Cephalalgia is becoming a much less frequent symptom, and when present is less severe. Sp. gr. of urine 1025.

Nov. 11th.—Has steadily improved since last date, In spite of the drawback of constant overwork and perplexity in business matters. Sp. gr. of urine to day, 1022—no excess of urea. He inhales 220 cubic inches of gas at an inspiration.

Nov. 20th.—Urine contains no abnormal ingredients—no excess of urea, Sp. gr. 1018. Patient considers himself mnch more nearly "cured" than he had supposed was possible. His digestion is very good, he sleeps well, and has lost nearly all his former irritability and inclination to despondency. His circulation is much more uniform—extremities in a comfortable glow most of the time, very little headache, and he does not become easily fatigued. Treatment suspended.

Jan. 5th.—Mr. S——— called and reports that he continues in every way improved.

